# Expression of Major Basic Protein and Endothelial Adhesion Molecules in Chronically Inflamed Mucosa of the Ethmoid Labyrinth

**DOI:** 10.1002/iid3.70066

**Published:** 2024-12-19

**Authors:** Tijana Vukadinović, Biserka Vukomanović Đurđević, Aleksandar Perić

**Affiliations:** ^1^ University of Montenegro Faculty of Medicine Podgorica Montenegro; ^2^ Clinic of Otorhinolaryngology, Clinical Center of Montenegro Podgorica Montenegro; ^3^ Institute for Pathology, Faculty of Medicine of the Military Medical Academy University of Defence Belgrade Serbia; ^4^ Department of Otorhinolaryngology, Faculty of Medicine of the Military Medical Academy University of Defence Belgrade Serbia

**Keywords:** eosinophils, immunohistochemistry, inflammation, nasal polyps, nasal surgical procedures

## Abstract

**Background/Objectives:**

Tissue remodeling, including dense eosinophil infiltration, is essential for forming inflammatory nasal polyps (NPs) and the pathogenesis of chronic rhinosinusitis with nasal polyps (CRSwNP). Toxic eosinophil major basic protein (MBP) damages the sinus mucosa epithelium and *lamina propria*, which initiates reparative processes leading to tissue remodeling. MBP specifically binds to BMK‐13 antibodies allowing immunohistochemical (IHC) tissue staining for eosinophils. This study evaluated the association between NP stromal BMK‐13 and endothelial adhesion molecule staining, and clinical parameters of NP patients compared to IHC expression and clinical parameters in subjects with healthy nasal mucosa.

**Methods:**

We included 30 patients with bilateral NPs who were selected for endoscopic ethmoidectomy. The control group was of 30 subjects with non‐inflamed nasal mucosa but with middle turbinate aeration, chosen for surgery. All participants were clinically scored before surgery, according to quality of life (QoL) outcome and symptoms. The degree of disease extension on computed tomography scans of the paranasal sinuses was also evaluated. Tissue samples after surgery were IHC stained for BMK‐13 and endothelial proliferation markers CD31 and CD34.

**Results:**

Expression of BMK‐13, CD31, and CD34 in tissues of NPs was higher than in healthy nasal mucosa. Positive correlations were observed between BMK‐13 expression, impaired QoL, and radiologically assessed extension of inflammation in NP patients.

**Conclusion:**

Apart from the fact that the NP tissue has, as expected, more intense eosinophilic infiltration, the proliferation of blood vessels is more pronounced in the NP tissue than in the tissue of healthy nasal mucosa. Expression of MBP in the tissue of ethmoidal NPs could serve as a potential marker of the degree of expansion of CRSwNP and indicate the severity of the disease.

**Clinical Trial Registration:**

None, because it was a cross‐sectional study.

## Graphical abstract

Expression of BMK‐13, CD31, and CD34 in tissues of NPs was higher than in healthy nasal mucosa. In patients With NP, we found a positive correlation between BMK‐13, impaired QoL, and radiologically evaluated disease extent. Apart From the fact that the NP tissue has, as expected, more intense eosinophilic infiltration, the proliferation of blood vessels is more pronounced in the NP tissue than in the tissue of healthy nasal mucosa. Expression of MBP in the tissue of ethmoidal NPs can serve as a potential marker of the degree of expansion of CRSwNP and may indicate the severity of the disease, implying the possibility that MBP could be a target molecule for future biological therapy of this disease.

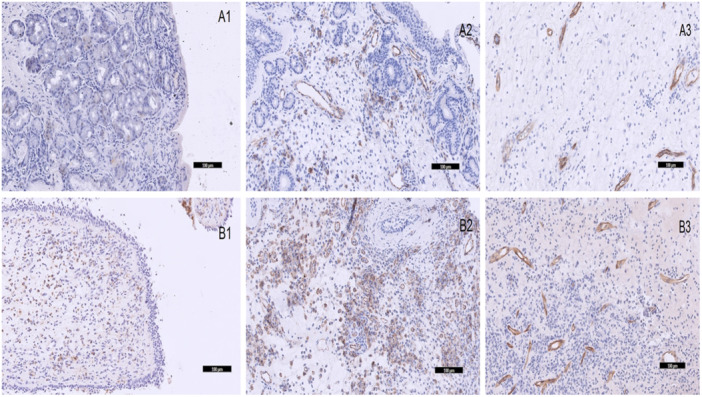

**Abbreviations**
AAO‐HNS: American Academy of Otolaryngology, and Head and Nech Surgery
BMK13: Eosinophil major Basic Protein antibody
CCAD: central compartment atopic disease
CD31: cluster of differentiation molecule 31
CD34: cluster of differentiation molecule 34
CRS: chronic rhinosinusitis
CRSwNP: chronic rhinosinusitis with nasal polyposis
ENT: ear, nose and throat
EPOS: European Position Paper on Rhinosinusitis and Nasal Polyps
LMS: Lund‐Mackay score
MBP: major basic protein
NP: nasal polyposis
NPs: nasal polyps
NSAIDs: non‐steroid anti‐inflammatory drugs
SNOT 22: Sino‐nasal Outcome Test 22
VAS: visual analog score

## 1 Introduction

Tissue remodeling of the nasal and paranasal sinuses mucosa is essential for forming inflammatory nasal polyps (NPs). The proliferation of respiratory epithelial cells and the thickening of the basement membrane are histological characteristics of chronic rhinosinusitis with nasal polyps (CRSwNP) or nasal polyposis (NP). At the same time, the stroma of NPs is dominated by strong edema and a dense inflammatory infiltrate [1, 2, 3]. NP in more than 90% of the world's population is characterized by a predominance of the T2 immune response and tissue eosinophilia [1, 2]. After being activated by T2 cytokines, such as interleukins 4, 5, and 13, eosinophils deposit toxic enzymes in their cytoplasmatic granules [4, 5]. Following the degranulation of eosinophils during chronic inflammation, these enzymes, primarily major basic protein (MBP), eosinophil cationic protein (ECP), and eosinophil peroxidase (EPO), damage the structure of the basal membrane, lamina propria and respiratory epithelium of the nasal/sinus mucosa, which initiates reparative processes leading to tissue remodeling [4, 5]. MBP specifically binds to BMK‐13 antibodies which allows immunohistochemical staining. In this way, activated eosinophils are detected in the pathohistological preparation [4]. Parallel to the eosinophil infiltration, the process of angiogenesis takes place in the stroma of CRS patients. Under the influence of growth and proangiogenic factors, immature blood vessels with increased vascular permeability are formed [6, 7]. This leads to the strong edema of the lamina propria, which is part of the tissue remodeling process. Cluster of differentiation (CD) molecules, CD31 and CD34 are reliable biomarkers of neoangiogenesis [6, 7, 8]. Both molecules are cell surface glycoproteins and members of the immunoglobulin superfamily. CD31, also known as platelet endothelial cell adhesion molecule 1 (PECAM‐1), is a pan‐endothelial marker. It is also an adhesion molecule that plays a role in the migration of leukocytes between endothelial cells, including eosinophils [9]. CD34 is also an intercellular adhesion molecule, expressed on fibroblasts, endothelium cells, and hematopoietic progenitor cells [10]. Both CD31‐ and CD34‐positive cells play roles in immune processes in the respiratory mucosa, including pathophysiological processes in the formation of NP [6, 7, 8, 9, 10].

Eosinophil stromal accumulation in NPs has been relatively well investigated. In contrast, neovascularization of the stromal layer in NPs was rarely studied [6, 7, 8, 9, 10]. Also, studies did not evaluate the potential relationship between stromal vascularization, eosinophil infiltration, and clinical characteristics of these patients. We hypothesized that there is an association between the MBP toxic effects on the sinus mucosa and the proliferation of new blood vessels as a part of tissue remodeling. So, this study aimed to compare the levels of stromal immunoexpression of BMK‐13, CD31, and CD34 in NPs and nasal mucosa without inflammation, as well as to investigate whether there is a correlation between the expression of these molecules and clinical parameters of NP patients.

## 2 Methods

### 2.1 Ethical Consideration

This cross‐sectional study was conducted according to the principles of human investigation that adhered to the Declaration of Helsinki. The Ethics Committee of our tertiary care hospital approved the protocol of this investigation (IRB Approval 21/2022). All patients signed an informed consent form for the study. The STROBE reporting method was used to present the methodology and results.

### 2.2 Patient Selection

The patients with NP, surgically treated and followed up between September 2020 and August 2023 in the Department of Otorhinolaryngology of the Military Medical Academy, were involved in this investigation. CRS was diagnosed following the EPOS 2020 [2] and the AAO‐HNS [11] guidelines. The patients with CRSwNP had the bilateral involvement of ethmoid labyrinth and they were without asthma and sensitivity to non‐steroid anti‐inflammatory drugs (NSAIDs). This limitation was introduced because patients with asthma and hypersensitivity to NSAIDs must take inhaled and systemic corticosteroid therapy due to the severity of their symptoms, which can affect the histological features of NP. Also, patients with central compartment atopic disease (CCAD), in which polyps mainly grow from the middle and upper nasal turbinate mucosa and in which the disease does not involve the paranasal sinuses, were excluded by ENT specialists after the computed tomography scan (CT) of the paranasal sinuses and nasal endoscopy. In these patients, the polyps are more centrally located, and less grow out of the middle nasal meatus.

Patients without nasal inflammation, but surgically treated in the same period of nasal obstruction due to pneumatization of the middle turbinate (*concha bullosa*) and deformation of the nasal septum were selected for the control group. They mostly reported chronic difficulty breathing through the nose, feeling fullness and pressure in the facial area, and headaches. Endoscopic examination revealed hypertrophy of the middle turbinate with or without deformation of the nasal septum, although there were no signs of inflammation of the nasal mucosa. Blood laboratory tests were normal. Skin prick and serological tests of hypersensitivity to inhaled allergens were negative. Cone beam computed tomography (CBCT) of the facial area showed pneumatization of the middle nasal concha and nasal septum deformation, without findings on the paranasal sinuses. Based on that, it was estimated that there is a need for surgical correction of the middle turbinate and nasal septum.

Subjects aged ≤ 18 and ≥ 65 years, pregnant and nursing women, patients with systemic diseases of the nasal cavity/sinuses, with other forms of inflammatory polyps (choanal polyp, hamartoma), fungal sinusitis, congenital disorder of the respiratory mucosa (primary ciliary dyskinesia, etc.), smokers, with previous surgery of the nose/sinuses, that use antihistamines and corticosteroids a month prior the start of the study, were excluded.

### 2.3 Clinical Assessment

All participants from the NP group and the control group were preoperatively clinically assessed by two ENT specialists. The intensity of nasal symptoms (nasal congestion—obstruction, increased nasal secretion, increased flow of secretion into the pharynx, headache, a feeling of fullness in the sinuses, weakened sense of smell) was evaluated by using a VAS score (from 0—no symptom to 10—maximum intensity of symptom) [12]. To assess the quality of life (QoL), we used the Sino‐nasal Outcome Test 22 (SNOT‐22) questionnaire in all participants [12]. Therefore, CRS patients were evaluated for the spreading of sinus disease on computed tomography (CT) scans of the paranasal sinuses under the Lund‐Mackay score (LMS) [13].

### 2.4 Tissue Sampling, Preparation, and Immunohistochemical Analysis

All tissue samples were collected during the surgical treatment. NP tissue samples were taken from the cells of the ethmoidal labyrinth during endoscopic sinus surgery (ESS). Tissue samples of the nasal mucosa in control subjects were taken by endoscopic resection of the lateral portion of the pneumatized middle concha and the pathohistological and immunohistochemical analysis included the mucosa lining the inside of the *concha bullosa*. Pathohistological and immunohistochemical analyses were performed by a pathologist who had no insight into the clinical characteristics of the participants. After staining the tissue specimens with hematoxylin and eosin, immunohistochemical analysis was performed. Human BMK‐13 antibody (Santa Cruz Biotechnology, Inc. Dallas, Texas, USA) was used to detect MBP‐positive eosinophils, while human anti‐CD31 and anti‐CD34 antibodies (Elabscience, Houston, Texas, USA) were used to detect CD31 and CD34 molecules. Analysis of immunohistochemical findings was performed with a digital light microscope. We used the morphometric Image Analysis Standard 1 (IAM‐1) software (Applied Image Inc, Rochester, NY, USA). We analyzed the entire slide and made an arithmetic mean from 10 fields of view with the densest cell distribution. The surfaces are measured automatically by software, not subjectively. We measured the number of marker‐positive cells in the whole slide. Immunoreactivity for BMK‐13 was evaluated based on the degree of binding of antibodies to MBP of eosinophils in the degranulation phase. Quantification of activated eosinophils was performed as follows: degree 0—no positive eosinophils, degree 1—few positive eosinophils (less than 5), degree 2—moderate number of eosinophils (5–10), degree 3—moderate number of grouped eosinophils (5–10 grouped eosinophils), degree 4—a large number of eosinophils, including groups of eosinophils (more than 10 single or grouped eosinophils) [4]. Immunoreactivity for CD31 and CD34 adhesion molecules was evaluated as the degree of staining of the cell membrane of endothelial cells in the continuity of the blood vessel lumen as follows: degree 0—the absence of staining, degree 1—weak staining, degree 2—moderate staining, and degree 3—strong staining [6]. All three IHC parameters were calculated using the formula: total number of eosinophils/blood vessels per examined area/subepithelial area (mm^2^). This gave us the number of BMK‐13 positive eosinophils and CD31 and CD34 positive blood vessels per square millimeter of tissue for a single sample [6].

### 2.5 Power of the Study and Sample Size Calculation

The power of the study needed to be at least 80% (0.8), and the probability of error of the first type (*α*) 0.05. Based on the data from the literature [4], BMK‐13 expression is expected to be highly significantly elevated in the patients with NP as compared to the control nasal mucosa (*p* < 0.001). According to the same paper, we assumed that the standard deviation (SD) values would be relatively high. A moderate effect size (0.34) was chosen to calculate the group size. Approximately 60 of the participants (30 in each group) were required to reach statistical significance at the *p* < 0.05 level between groups. We used the analysis of variance test (analysis of Variance, fixed effects, omnibus, one‐way) with commercial software (GPower 3.1.).

### 2.6 Statistical Analysis

The authors used the non‐parametric Mann–Whitney *U* test to compare parameters between patient groups. To evaluate differences in the intensity of clinical and biochemical parameters between groups, we used the Chi‐square test and Fisher's exact test. The degree of correlation between parameters was assessed using the Spearman coefficient of correlation. A *p *< 0.05 is considered statistically significant. Results related to clinical parameters in the figures and tables are represented as mean ± standard deviation (SD).

## 3 Results

During the period in which we conducted research, 58 patients with polypoid inflammatory growths in the nose and paranasal sinuses were registered. From that number, 10 patients with choanal polyps, 9 patients with CCAD, 6 patients with fungal sinusitis, and 3 patients with nasal hamartomas were excluded. Also, 42 patients with *concha bullosa* were registered, but 12 patients were excluded due to signs of inflammation of the nasal mucosa, whether they were infectious or allergic inflammation. A total of 60 patients were included in the present study: NP patients (*n* = 30), and a control group consisting of patients without inflammatory disease, but with chronic nasal obstruction due to pneumatization of the middle turbinate (*n* = 30). No statistical difference was found in the mean age and gender distribution of patients between two patient groups (*p* > 0.05; *p* > 0.05, respectively). Results related to demographic data are presented in Table 1.

**Table 1:** Demographic parameters.

Parameters	Nasal polyposis	Controls	*p* value
Patients	30	30	> 0.05
Age*/years	44.8 ± 13.1	46.7 ± 13.8	> 0.05
Men/Women	16/14	15/15	> 0.05

*Note:* *Means that this parameter (Age*/years) is expressed as mean +/− SD (standard deviation).

**Figure 1:**
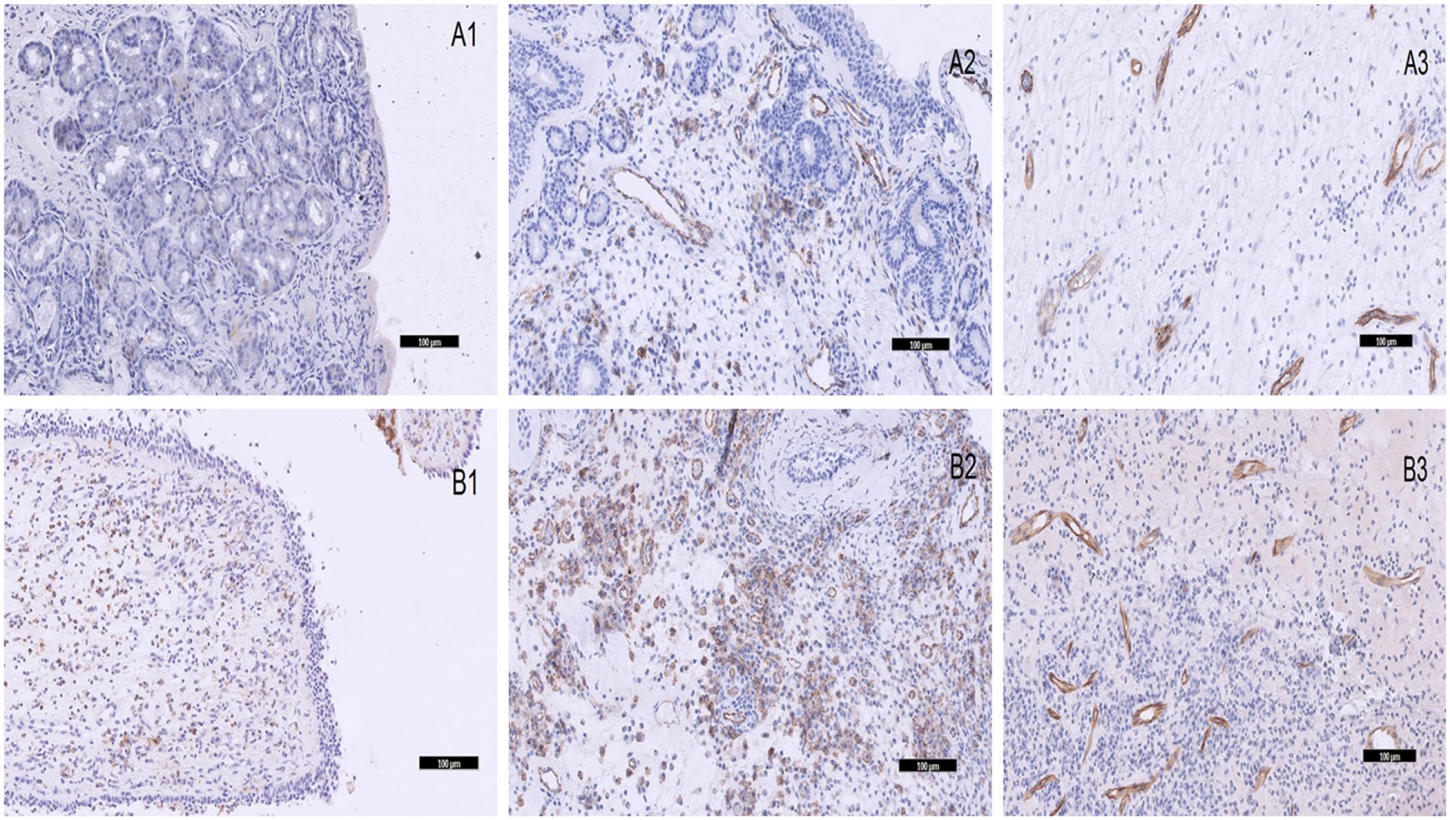
Examples of immunohistochemical staining in the *concha bullosa* (CB) mucosa (top row) and nasal polyps (NPs) (bottom row). A1, BMK‐13 expression in the CB mucosa; A2, CD31 expression in the CB mucosa; A3, CD34 expression in the CB mucosa; B1, BMK‐13 expression in NPs; B2, CD31 expression in NPs; B3, CD34 expression in NPs. (Magnification ×100). On slide A1, we can see zero degrees, and on slide B1, we can see degree 4 of immunoreactivity for BMK‐13. On slide A2, we can see the 1st degree, and on slide B2, we can see degree 3 of immunoexpression for CD31. On slide A3, we can see the 2nd degree, and on slide B3, we can see degree 3 of immunoreactivity for CD34.

We found higher VAS and SNOT‐22 scores in NP patients than in participants without nasal inflammation (*p* = 0.008; *p* < 0.001, respectively). Examples of immunohistochemical staining for two groups of patients are shown in Figure 1. We did not find a complete absence of CD31 and CD34 immunoreactivity in any subjects. Concerning CD31 and CD34, the higher frequency of the strongest staining (degree 3) was observed in tissue samples obtained from NP patients than in controls (*p* = 0.018; *p* = 0.026, respectively). The eosinophils were mostly localized just below the basement membrane and around the vessels and glands. BMK‐13 expression was very low in the control group, while in NP patients we found high levels of BMK‐13 staining. Levels of immunoreactivity for BMK‐13 were highly statistically higher for eosinophil infiltration grades 1, 2, 3, and 4 in patients with NP compared to controls (*p* = 0.006; *p* < 0.001; *p* < 0.001; *p *< 0.001, respectively). Numerical data of clinical and immunohistochemical parameters are presented in Table 2.

**Table 2:** Clinical and immunohistochemical parameters in NP patients and controls.

Parameters	Nasal polyposis	Controls	*P* value
VAS^a^	6.5 ± 1.8	4.2 ± 1.8	0.008
SNOT‐22^a^	48.8 ± 12.9	28.7 ± 18.5	< 0.001
LMS^a^	15.7 ± 4.6	0	/
BMK‐13 expression	0 – 6.4%	0 – 88.5%	< 0.001
	1 – 26.7%	1 – 7.1%	0.006
	2 – 34.5%	2 – 3.8%	< 0.001
	3 – 19.3%	3 – 0.6%	< 0.001
	4 – 13.1%	4 – 0.0%	< 0.001
CD34 expression	1 – 22.5%	1 – 32.4%	0.028
	2 – 41.7%	2 – 37.3%	> 0.05
	3 – 35.8%	3 – 30.3%	0.026
CD31 expression	1 – 27.2%	1 – 48.7%	0.004
	2 – 43.4%	2 – 35.7%	0.036
	3 – 29.6%	3 – 15.6%	0.018

*Note:* The degrees of immunoreactivity for BMK‐13 were expressed as 0, 1, 2, 3, and 4, whereas for CD31/CD34 were expressed as 0, 1, 2, and 3. There was not a single sample with negative expression for CD31 and CD34.

Abbreviations: LMS, Lund‐Mackay score; SNOT‐22, Sino‐nasal Outcome Test‐22; VAS, visual analog score.

^a^ Results are presented as mean ± SD (standard deviation).

Regarding the correlations, the present study showed a moderate positive correlation between BMK‐13 and SNOT‐22 (*r* = 0.48) and between BMK‐13 and LMS (*r* = 0.54) in NP patients (Table 3). In the control group, we found a positive correlation between CD31 and CD34 (*r* = 0.43) (Table 4).

**Table 3:** Correlations between immunohistochemical markers and clinical score parameters in patients with NP.

Parameters	VAS	SNOT‐22	LMS	CD31	CD34	BMK‐13
VAS	/	*r* = 0.32	***r*** = **0.53** **	*r* = 0.25	*r* = 0.17	*r* = 0.31
		*p* > 0.05	***p*** = **0.007**	*p* > 0.05	*p* > 0.05	*p* > 0.05
SNOT‐22	*r* = 0.32	/	***r*** = **0.56**	*r* = −0.13	*r* = 0.083	***r*** = **0.48** **
	*p* > 0.05		***p*** < **0.001** ***	*p* > 0.05	*p* > 0.05	***p*** = **0.005**
LMS	***r*** = **0.53** **	***r*** = **0.56** ***	/	*r* = 0.16	*r* = 0.061	***r*** = **0.54** ***
	***p*** = **0.007**	***p*** < **0.001**		*p* > 0.05	*p* > 0.05	***p*** < **0.001**
CD31	*r* = 0.25	*r* = −0.13	*r* = 0.16	/	*r* = 0.16	*r* = 0.11
	*p* > 0.05	*p* > 0.05	*p* > 0.05		*p* > 0.05	*p* < 0.05
CD34	*r* = 0.17	*r* = 0.083	*r* = 0.061	*r* = 0.16	/	*r* = 0.15
	*p* > 0.05	*p* > 0.05	*p* > 0.05	*p* > 0.05		*p* > 0.05
BMK‐13	*r* = 0.31	***r*** = **0.48** **	***r*** = **0.54** ***	*r* = 0.11	*r* = 0.15	/
	*p* > 0.05	***p*** = **0.005**	***p*** < **0.001**	*p* > 0.05	*p* > 0.05	

*Note:* Values in the boxes (*r*) represent the Spearman's correlation coefficient.

Abbreviations: LMS, Lund‐Mackay Score; SNOT‐22, Sino‐nasal Outcome Test‐22; VAS, visual analog score.

* *p* < 0.05;

** *p* < 0.01;

*** *p* < 0.001.

**Table 4:** Correlations between immunohistochemical markers and clinical score parameters in subjects from the control group.

Parameters	VAS	SNOT‐22	CD31	CD34	BMK‐13
VAS	/	***r*** = **0.69** ***	*r* = 0.23	*r* = 0.022	*r* = 0.30
		***p*** < **0.001**	*p* > 0.05	*p* > 0.05	*p* > 0.05
SNOT‐22	***r*** = **0.69** ***	/	*r* = 0.078	*r* = −0.054	*r* = 0.087
	***p*** < **0.001**		*p* > 0.05	*p* > 0.05	*p* > 0.05
CD31	*r* = 0.23	*r* = 0.078	/	***r*** = **0.43** **	*r* = 0.18
	*p* > 0.05	*p* > 0.05		***p*** = **0.007**	*p* > 0.05
CD34	*r* = 0.022	*r* = ‐0.054	**r** = **0.43** **	/	*r* = 0.26
	*p* > 0.05	*p* > 0.05	***p*** = **0.007**		*p* > 0.05
BMK‐13	*r* = 0.30	*r* = 0.087	*r* = 0.18	*r* = 0.26	/
	*p* > 0.05	*p* > 0.05	*p* > 0.05	*p* > 0.05	

*Note:* Values in the boxes (*r*) represent the Spearman's correlation coefficient.

Abbreviations: SNOT‐22, Sino‐nasal Outcome Test‐22; VAS, visual analog score.

* *p* < 0.05

** *p* < 0.01

*** *p* < 0.001.

## 4 Discussion

In this study, we compared the histological characteristics of NPs removed from the ethmoidal labyrinth and the non‐inflamed inner mucosa of the pneumatized middle nasal concha. In the vast majority of patients with bilateral CRSwNP, inflammatory nasal polyps primarily form in the area of the ethmoid labyrinth, including the ostiomeatal complex [1, 2]. Aeration of the middle turbinate is one of the most common anatomical variants in the sino‐nasal region and is represented in 17%–36% of the human population [14]. In most cases, *concha bullosa* originates from the anterior ethmoid cells and the middle nasal meatus [14]. Apart from the fact that the middle turbinate embryologically belongs to the ethmoidal region, there are other reasons why we did not take control samples of the nasal mucosa from the inferior turbinate. Patients with CRSwNP exhibit a significant disorder in immunomodulatory mechanisms, as well as abnormal tissue remodeling processes. However, the production of transforming growth factor beta (TGF‐β), one of the main drivers of tissue remodeling is reduced in NP, which conditions the reduced production of collagen compared to the healthy nasal mucosa [15]. The altered composition of the extracellular matrix in NP leads to mechanical dysfunction in the nasal/sinus mucosa and reduced interstitial hydrostatic pressure during extravasation of plasma through newly formed immature blood vessels into the tissue of NPs [15]. Pezato et al. [16] found a higher increase of interstitial hydrostatic pressure during the saline injection in the mucosa of the middle than in the mucosa of the inferior turbinate. Thus, in the stroma of the mucous membrane of the middle turbinate, there is significantly more space for the accumulation of interstitial fluid compared to the stroma of the inferior turbinate. For this reason, it is better to histologically compare the middle turbinate mucosa with inflammatory NPs.

NP is usually histologically characterized by dense eosinophilic infiltration, but in a large number of these patients, eosinophilia is also present in the blood and is a predictor of the severity of the disease and the tendency to relapse [17]. Considering the predominance of the T2 immune response, the therapy of choice is, apart from surgery, local and/or systemic corticosteroid use, which depends on the extent of the disease and the severity of NP symptoms [18].

New blood vessel formation accompanies chronic inflammation in the nasal/sinus mucosa during tissue remodeling. Several inflammatory mediators released from immune response cells are signaling molecules or stimulate the production of angiogenesis signaling molecules, including cytokines, chemokines, adhesion molecules, and growth factors [19, 20]. These proangiogenic mediators stimulate endothelial cells to proliferate and form new blood vessels [20]. Previous research has shown that the expression of CD34 molecules is higher in the tissue of bilateral NPs compared to antrochoanal polyps and healthy nasal mucosa [10]. In addition, CD34 is higher in the tissue of NPs from patients with a dominant T2 immune response compared to the tissue of NPs from patients with a dominant T1 immune response [21]. These results suggest an association between the eosinophilic inflammation that predominates in patients with T2 mediator‐driven CRS and endothelial proliferation in the stroma. Also, a higher expression of proangiogenic genes resulting in a higher local expression of CD31 (PECAM) was observed in patients with CRSwNP compared to healthy subjects [22].

The results of the present study showed better expression of all three markers in the NP tissue than in the tissue of non‐inflamed nasal mucosa. The composition of eosinophils includes previously formed enzymatic and nonenzymatic cationic proteins, which are selectively secreted from their large granules [23]. These proteins contribute to tissue damage and airway mucosal remodeling including smooth muscle hyperplasia and subepithelial fibrosis [23]. The MBP is an arginine‐rich protein. Studies of MBP mRNA have shown that MBP is synthesized from the preform. Eosinophil granule MBP‐1 forms the core of secondary eosinophil granules [23, 24]. Recently, a less cationic homolog of MBP, called MBP‐2 has been discovered [24]. Immunofluorescence demonstrated that MBP‐1 is present in the eosinophils, basophils, and mast cells, while MBP‐2 is detected only in eosinophils. Neither MBP‐1 nor MBP‐2 can be detected in any other peripheral blood white cells [24]. MBP causes the accumulation of sodium and chlorine ions which results in the accumulation of water molecules in the intercellular space and the formation of edema [23, 24]. The presence of activated eosinophils results in damage to the epithelium of the nasal mucosa [23, 24]. However, their influence on subepithelium primarily on blood vessels, has not been sufficiently investigated. In our study, significantly higher expression of eosinophilic infiltration marker BMK‐13 is fully expected, but, interestingly the expression of angiogenic markers CD31 and CD34 is also higher in NP tissue. The healthy nasal mucosa is better supplied with blood compared to the tissue of NPs, but the formation of young blood vessels is more intense in the tissue of NPs. The positive correlation of BMK‐13 expression with clinical parameters of NP patients reflects the fact that the intensity of eosinophilic infiltration is directly related to the extent of the disease. The absence of correlation between the expression of BMK‐13 and vascular adhesion molecules CD31 and CD34 is at first glance an unexpected finding. However, although CD31 and CD34 adhesion molecules are important for the process of passing eosinophils between endothelial cells before their accumulation in the tissue, they are not dominant in this role [6, 7, 8, 9, 10, 25]. Several other adhesion molecules play an important role in the transmigration of leukocytes, including eosinophils, through capillary walls. Intercellular adhesion molecule 1 (ICAM‐1), ICAM‐2, vascular cell adhesion molecule 1 (VCAM‐1), and mucosal addressin cell adhesion molecule 1 (MAdCAM‐1) are just some of them. A key role in the process of such transendothelial migration of eosinophils is played by vascular cell adhesion molecule 1 (VCAM‐1), whose production in NP fibroblasts is stimulated by T2 cytokines, especially IL‐4, as well as by strong pro‐inflammatory cytokine tumor necrosis factor‐alpha (TNF‐α) [26]. Without the influence of VCAM‐1, the accumulation of eosinophils in the mucosal stroma would be significantly impaired [26]. This data implies the need for further investigation of the association between immunoexpression of MBP and VCAM‐1 in NP tissue.

Our study has some limitations. Due to the necessity of frequent use of inhaled and systemic corticosteroids, we did not include NP patients with asthma and NSAID hypersensitivity. Patients with CCAD, although it is considered a form of CRSwNP associated with allergic rhinitis, were excluded from the study, considering that the polypoid degeneration affects mainly the mucosa of the middle and/or upper nasal concha, and the changes on CT scans are less pronounced. Local potential for the production of MBP and endothelial adhesion molecules is better assessed based on gene expression, which we did not do due to financial constraints. Thus, we recommend further research using reverse transcription polymerase chain reaction (RT‐PCR) and other gene expression studies to assess these products and to delineate any gene mutation that causes the overexpression of these protein products as not all CRS patients develop NP.

## 5 Conclusion

The results of our study showed a weak presence of MBP‐releasing eosinophils in the healthy middle turbinate mucosa. On the other hand, the expression of BMK‐13 is extremely high in the NP tissue. Markers of endothelial proliferation are also significantly more expressed in NP tissue. The intensity of eosinophilic infiltration in the *lamina propria* correlates with the radiologically estimated extent of the disease and the impairment of QoL. Thus, the expression of MBP in the tissue of ethmoidal NPs could serve as a potential marker of the degree of expansion of CRSwNP and may indicate the severity of the disease.

## Author Contributions

**Tijana Vukadinović:** conceptualization, data curation, formal analysis, investigation, methodology, visualization, writing–original draft, writing–review and editing. **Biserka Vukomanović Đurđević:** data curation, formal analysis, investigation, methodology, resources, supervision, validation, visualization, writing–review and editing. **Aleksandar Perić:** conceptualization, data curation, formal analysis, funding acquisition, investigation, methodology, project administration, resources, supervision, validation, visualization, writing–original draft, writing–review and editing.

## Ethics Statement

All procedures performed in studies involving human participants were under the ethical standards of the institutional and/or national research committee and with the 1964 Helsinki Declaration and its later amendments or comparable ethical standards. Ethics committee approval was received for this study from the Ethics Committee of the Military Medical Academy, Belgrade, Serbia (MMA No 21/2022) in June 01, 2022. Written informed consent was obtained from all individual participants included in the study.

## Conflicts of Interest

The authors declare no conflicts of interest.

## Data Availability

The data sets used/analyzed during the current study are available from the corresponding author upon reasonable request.
